# Exergames versus self-regulated exercises with instruction leaflets to improve adherence during geriatric rehabilitation: a randomized controlled trial

**DOI:** 10.1186/s12877-017-0467-7

**Published:** 2017-03-23

**Authors:** Peter Oesch, Jan Kool, Luis Fernandez-Luque, Ellen Brox, Gunn Evertsen, Anton Civit, Roger Hilfiker, Stefan Bachmann

**Affiliations:** 1 Rehabilitationsklinik Valens, Taminaplatz 1, 7317 Valens, Switzerland; 2 Norut, Tromsø, Norway; 3 Qatar Computing Research Institute, Hamad Bin Khalifa University, Qatar Foundation, Doha, Qatar; 4 Architecture and Computer Technology Department, University of Seville, Seville, Spain; 5 University of Applied Sciences and Arts Western Switzerland Valais (HES-SO Valais-Wallis), 1950 Sion, Switzerland; 6 Department of Geriatrics, Inselspital, Bern University Hospital, University of Bern, 3010 Bern, Switzerland

**Keywords:** Geriatric rehabilitation, Older adults, Self-regulated exercise, Exergames, Adherence, Motivation, Mobility

## Abstract

**Background:**

Improving mobility in elderly persons is a primary goal in geriatric rehabilitation. Self-regulated exercises with instruction leaflets are used to increase training volume but adherence is often low. Exergames may improve adherence. This study therefore compared exergames with self-regulated exercise using instruction leaflets. The primary outcome was adherence. Secondary outcomes were enjoyment, motivation and balance during walking.

**Methods:**

Design: single center parallel group non-blinded randomized controlled trial with central stratified randomization. Setting: center for geriatric inpatient rehabilitation. Included were patients over 65 with mobility restrictions who were able to perform self-regulated exercise. Patients were assigned to self-regulated exercise using a) exergames on Windows Kinect® (exergame group EG) or b) instruction leaflets (conventional group CG). During two 30 min sessions physical therapists instructed self-regulated exercise to be conducted twice daily during thirty minutes during ten working days. Patients reported adherence (primary outcome), enjoyment and motivation daily. Balance during walking was measured blind before and after the treatment phase with an accelerometer. Analysis was by intention to treat. Repeated measures mixed models and Cohen’s d effect sizes (ES, moderate if >0.5, large if > 0.8) with 95% CIs were used to evaluate between-group effects over time. Alpha was set at 0.05.

**Results:**

From June 2014 to December 2015 217 patients were evaluated and 54 included, 26 in the EG and 28 in the CG. Adverse effects were observed in two patients in the EG who stopped because of pain during exercising. Adherence was comparable at day one (38 min. in the EG and 42 min. in the CG) and significantly higher in the CG at day 10 (54 min. in the CG while decreasing to 28 min. in the EG, *p* = 0.007, ES 0.94, 0.39–0.151). Benefits favoring the CG were also observed for enjoyment (*p* = 0.001, ES 0.88, 0.32 – 1.44) and motivation (*p* = 0.046, ES 0.59, 0.05–1.14)). There was no between-group effect in balance during walking.

**Conclusions:**

Self-regulated exercise using instruction leaflets is superior to exergames regarding adherence, enjoyment and motivation in a geriatric inpatient rehabilitation setting. Effects were moderate to large. There was no between group difference in balance during walking.

**Trial registration:**

ClinicalTrials.gov, NCT02077049, 6 February 2014.

## Background

### Mobility and physical activity

The proportion of persons over 65 years of age in Europe is increasing significantly [1]. Aging is associated with a decline in mental function, reduced motivation for physical activities, a decline in motor skills, mobility impairment and a higher risk of falling [2]. Recommendations for persons over 65 years of age include aerobic physical activity [3], and strengthening and balance exercises several times a week [4], which have been shown to reduce age-related decline, institutional placement and mortality [5, 6].

### Self-regulated exercise and exergames

In order to increase the quantity and duration of therapy during rehabilitation, self-regulated exercise is prescribed in addition to supervised sessions. However, self-regulated exercise programs using instruction leaflets are often considered boring [7]. Exergames may be an attractive alternative for increasing the motivation of elderly people performing self-regulated exercises. Exergames are designed for a primary purpose other than pure entertainment. During exergames the user performs physical exercises to control the game. Exergames rely on technology that tracks body movement and reaction, and are designed to promote an active lifestyle by using persuasive technology [8–10]. A common barrier is usability, since elderly users are often not familiar with computer technology [11–13]. Furthermore, for geriatric rehabilitation purposes, these games must be task-oriented and closely map real-world activities [14]. Instant feedback, social play and personalization, improve their persuasiveness [15].

### Effectiveness of exergames

There is limited evidence regarding the effectiveness of exergames in increasing adherence to exercise recommendations in rehabilitation settings. A review of exergames for stroke rehabilitation found moderate improvements in activity outcomes and highlighted the need for larger studies. The median study size was 11 participants per group and the largest study included 40 participants [16]. Other reviews of the efficacy of exergames for promoting physical activity in older adults also emphasize the need for more robust studies in order to determine the benefit of exergames [17–20]. Only seven of 56 studies in a recent review [19] compared the adherence to the exercises between exergames and a control group. Of these, four showed no difference and three showed better adherence in the exergaming group. The number of participants in those studies ranged from 17 to 65.

The present study was conducted within the GameUp project [21], which addressed these specific challenges by developing new exergames promoting mobility in elderly people, using well-documented user-centered design methods. Approximately 100 senior citizens and health experts participated in the design of an exergame called FarmUp.

### Aim

The primary aim of this study in a geriatric rehabilitation setting was to compare adherence to self-regulated training when playing exergames or using information leaflets for self-regulated exercises. Secondary outcomes enjoyment, motivation and balance skills.

## Methods

This clinical study was registered on ClinicalTrials.gov (identifier number: NCT02077049). The design and treatments used in this study have been described in more detail previously [22].

### Design

**Fig. 1:**
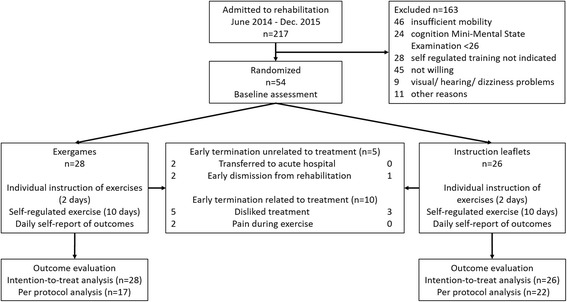
Study flowchart

A single-center randomized controlled clinical trial was performed comparing self-regulated conventional exercises with self-regulated exergames at Walenstadtberg Rehabilitation Clinic in Switzerland. Many of its clientele are persons over 65 years of age with musculoskeletal impairment (due to ortho-traumatology, internal medicine, oncology, or pulmonology) who are referred for inpatient rehabilitation from acute hospitals or by general practitioners. Patients were allocated by central randomization to the exergame group or to the conventional exercise group in a ratio of 1:1. Randomization was stratified according to balance and computer skills [22]. A research assistant collected patient ratings during the intervention phase and performed clinical assessments pre- and post-intervention. Figure 1 shows the study design and patient flow.

### Participants and recruitment

All patients over 65 years of age referred for inpatient rehabilitation from June 2014 to December 2015 were evaluated for inclusion in the study. Following medical screening by the doctor, patients were checked for inclusion and exclusion criteria by the study researcher. Inclusion criteria were: ability to walk independently over 20 m (with or without walking aids) and written informed consent. Exclusion criteria were: disorders limiting the use of computer games (e.g. neurological disorders, visual impairment, deafness, vertigo, cognitive impairment), or specific medical contra-indications, such as open wounds or severe pain, preventing prescription of self-regulated training. For the purpose of this study, cognitive impairment was defined as a Mini-Mental State Examination (MMSE) score <26 [23].

### Randomization and blinding

An independent investigator who was not involved in the trial generated a randomization schedule with four strata according to computer and balance skills using blocks of two. A research assistant blinded to the randomization schedule checked inclusion criteria in eligible patients, asked for written informed consent, and informed the therapy secretariat about the included patient and stratum according to computer and balance skills. The therapy secretariat performed randomization according to the schedule and planned patient treatments according to group assignment.

Participants and physiotherapists involved in the study were inevitably aware of treatment allocation. Due to the small size of the rehabilitation center, it was impossible to blind the study researcher to the treatment allocation over the whole trial period. However, the therapists, the study researcher and the patients were not able to influence the pre- and post-intervention measurements. The ActiGraph^®^ mobility tracker recorded and transmitted all data directly to the computer in an encrypted form. An external researcher, blinded to participants’ group allocation, analyzed the ActiGraph^®^ data.

Measurements of training volume, recorded in the logbook, and of enjoyment and motivation were patient-reported. Although patient-reported outcomes may be biased, systematic errors are likely to be comparable in both groups. Therefore, we considered between-group comparisons based on these self-reported outcomes to be valid.

### Interventions

All study participants were allocated two time-slots (2 × 30 min/d) from Monday to Friday, dedicated to self-regulated training, conventional or exergames, for ten working days. This was communicated to patients via a printed weekly therapy program containing the various medical appointments and therapy sessions for each day. Before the intervention started, all patients underwent two instruction sessions with a trained physiotherapist on how to perform the self-regulated training (conventional or exergames). Patients were instructed to repeat self-regulated exercises, conventional or exergames, during the allocated time-slots as many times as possible. In addition, all patients were encouraged to walk and climb stairs instead of using the elevator. This protocol ensured that all participants received the same attention at the beginning of the study and were equally motivated to perform self-regulated training (Fig. 1).

Exercises in both groups (conventional or exergames) aimed to improve balance, strength and mobility, based on the same physiological assumptions about, and physical requirements of, elderly people. Affordance levels of the exergames were therefore comparable to the conventional exercises.

### Safety

In order to ensure safety during self-regulated training, different levels of exercise difficulty were developed for both the conventional and the exergames programs. The physiotherapist selected appropriate exercises to tailor the exercise program to the patient’s balance skills, as assessed with the Berg Balance Scale (BBS). A BBS score <45 indicates a risk of falling, and thus those patients performed self-exercise in a sitting position only. Patients scoring between 45 and 55 points performed the exercises in a static standing position, whereas patients reaching the maximum score of 56 performed exercises in a dynamic standing position. Furthermore, a therapist assistant was always present during the scheduled exercise sessions to offer assistance if needed and to record adverse events.

### Conventional self-regulated exercises

Conventional self-regulated exercises using instruction leaflets are routinely prescribed at Walenstadtberg Rehabilitation Clinic to all suitable inpatients, with the objective of improving balance, strength and mobility. Exercises were performed in sitting, standing or walking, depending on the patient’s balance skills. All patients performed individual exercises, adapted to their balance abilities, within the gymnastics room of the clinic, according to a printed instruction sheet. Most of the time, several patients attended conventional self-regulated exercise sessions at the same time.

### Exergames

The GameUp project [21] developed seven mini-games for balance, leg strength and flexibility with a user-centered approach, making sure that elderly users could not only perform the exercises, but could also read, see and enjoy the graphics and sounds of the game. Mini-games were combined into three different exercise levels, performed in sitting, standing or walking, thereby allowing affordance adjustments according to the balance abilities of the individual player. The total length of the exercise program was 12 min. Players exercised in a special room and they were asked to repeat the program during the allocated 30 min exercise time. Termination of the program was possible at any time. In case of technical problems, a therapy assistant was available to help.

### Outcome measures

#### Primary outcome

The primary outcome of this randomized controlled trial was adherence, defined as the duration of daily self-regulated training in minutes. Patients recorded the duration of self-regulated exercise during each session in minutes and the number of sessions per day. We considered using the log data from the Kinect-based game to measure daily training volume in the exergames group. However, these measurements could not be used in the conventional exercise group. Therefore, we decided to use a standardized logbook with self-reported measures in both groups. The feasibility of the logbook was tested in a pilot study comparing conventional balance training with Nintendo Wii balance games in patients with stroke [24].

### Secondary outcomes

Secondary outcomes were motivation and enjoyment for each training day, and objective balance skills of the participants. After each training patients rated motivation and enjoyment on a five-level Likert scale, ranging from 1 = very low to 5 = very high, in the logbook. Objective dynamic balance (local dynamic stability) skills were assessed at pre- and post-intervention with a tri-axial ActiGraph accelerometer, size 4.6 × 3.3 × 1.5 cm, weight 19 g (ActiGraph LLC, Pensacola, FL 32502, USA [25]). The accelerometer measures trunk acceleration in medio-lateral, vertical and antero-posterior directions. The sampling rate was 100 Hz. The accelerometer was attached to the participants’ lower back at the level of the third lumbar vertebra and they were instructed to walk as fast as safely possible along a 50-m corridor.

### Sample size

The sample size calculation was based on a previous feasibility study [24] evaluating motivation and time spent in self-regulated exercise using exergames. Assuming a medium effect size (d = 0.5), a statistical power of 0.80, and a type I error risk of 0.05, a sample size of 64 subjects per group would be needed for between-group comparison with two measurement points. Although repeated measures with 10 time-points reduce the required sample size, we did not perform a sample size calculation for repeated measures because these are very unreliable due to the difficulty of estimating covariance between repeated measures a priori.

### Statistical analysis

Descriptive statistics were recorded at baseline for the two groups. Missing outcome data in patients who disliked treatment were substituted by lowest values. Missing data unrelated to treatment were substituted using expectation maximization including residual error. The assumption of normality was tested for the outcomes by visual inspection of histograms with a normality curve. Mixed model analysis was used for repeated-measures to account for the dependency of repeated measures within patients. Models included fixed effects for the interaction between group and time, time and group and random intercepts. A homogeneous autoregressive (order 1) or diagonal covariance structure was used, depending on the model fit evaluated, with -2log likelihood. As the aim of the study was to compare outcomes between groups during the 10-day treatment phase, hypotheses testing focused on the interaction between group and time. Cohen’s d effect sizes were computed using bootstrapped means and SDs of the first and last measurement and were considered small if >0.2, moderate if >0.5 and large if >0.8 [26]. Analyses were performed with a critical value of 0.05 using SPSS Version 23.

The three-dimensional acceleration data collected with the accelerometer ActiGraph^®^ was used to calculate the largest Lyapunov exponent (λS, “divergence exponent”). The λS is a non-linear gait stability index, which has been advocated as an early indicator of the risk of falls [25]. Lower values of λS indicate better stability. The peak in the acceleration signal corresponds to the heel strike in the gait cycle. After graphical inspection of the acceleration signal, 60 consecutive steps were selected for analysis. Data analysis was performed with the package tseriesChaos in R [27].

## Results

### Recruitment and baseline characteristics

During the recruitment period June 2014 to December 2015 a total of 217 patients were evaluated for inclusion. Of these, 54 (25%) were included and 163 (75%) excluded (Fig. 1).

The main reasons for exclusion were: insufficient mobility interfering with safety during exercise (21% of all eligible patients), not willing to participate in the study because patients disliked exercising using a computer game (20% of all eligible patients), and reduced cognition (11% of all eligible patients). Groups were comparable at baseline (Table 1).

**Table 1:** Baseline characteristics of the study population

	GameUp *N* = 26	Control *N* = 28	Between-group difference *p*-value
Age, median (IQR)	73.8 (67.9–79.1)	74.3 (66.1–79.3)	0.74^a^
Gender female, N (%)	9 (35)	16 (57)	0.11^b^
Diagnosis, N (%)			
Musculoskeletal post-surgery	12 (46)	12 (43)	0.75^b^
Musculoskeletal	8 (31)	6 (21)	
Internal medicine post-surgery	4 (15)	7 (25)	
Internal medicine	2 (8)	3 (11)	
Multimorbidity CIRS 14–56, median (IQR)	11 (7.3–16.5)	11 (7.0–13.3)	0.42^a^
Cognition MMSE 0–30, median (IQR)	28 (28–29)	28 (28–30)	0.65^a^
Balance BBS 0–56, median (IQR)	49 (37.5–53.0)	48 (36.50–53.2)	0.99^a^
Fear of falling FES, median (IQR)	21.0 (17.2–24.7)	21.5 (17.0–27.0)	0.92^a^
Walking balance (mean Lyapunov, SD)	1.431 (0.221)	1.395 (0.157)	0.70^c^

*IQR* interquartile range, *CIRS* Cumulative Illness Rating Scale, *MMSE* Mini-Mental State Examination, *BBS* Berg Balance Scale, *SD* standard deviation, *FES* Falls Efficacy Scale

^a^Mann-Whitney *U* test; ^b^χ^2^ test; ^c^independent samples *t*-test

### Dropouts and substitution of missing data

Patients completed a mean of 85% of the scheduled treatment sessions. No falls occurred. The median number of sessions was 15.5 (IQR 7.5–18) in the exergame group and 18.5 (IQR 12.75–25) in the instruction leaflet group). Early termination occurred in 15 patients (Fig. 1). Termination was unrelated to treatment in five patients, four in the exergame group after 10.5 sessions (IQR 7.5–15.0) and one in the control group after 10 sessions. Early termination was related to treatment in ten patients. Most of these patients stopped participation during the 2-day instruction phase. More patients in the exergame group compared to the control group (five versus two) disliked treatment or stopped because of pain during exercise (two versus zero). Seven patients in the exergame group stopped after a median of zero sessions (IQR 0-4) and three in the instruction leaflet group after zero sessions (IQR 0-1).

Missing outcome data in patients who disliked treatment and prematurely terminated participation were substituted by the lowest values. Missing values for adherence, assessed as minutes spent performing self-regulated exercise, were set at zero. Missing data for enjoyment and motivation were substituted by the lowest value (not experiencing enjoyment or feeling motivated at all). In patients who terminated earlier for reasons unrelated to treatment, missing data were substituted by expectation maximization including residual error.

### Outcomes

**Fig. 2:**
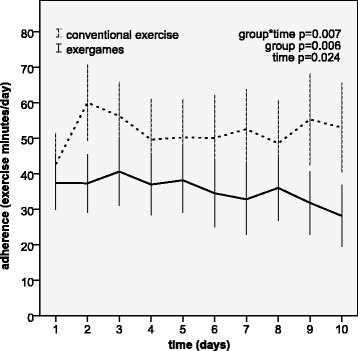
Adherence to prescribed self-regulated exercise (min/d) for 10 days of treatment

The primary outcome of the study was adherence to prescribed self-regulated exercise evaluated by self-reported exercise time (Fig. 2). There was a significant interaction between group and time favoring the conventional exercise group. The effect was large, as indicated by the effect size of 0.94 (95% confidence interval (95% CI) 0.39–0.151). Adherence was comparable in the two groups only on the first day, and gradually decreased in the exergame group, while it increased in the conventional exercise group. Adherence was generally higher in the conventional exercise group, as shown by the significant group effect.

**Fig. 3:**
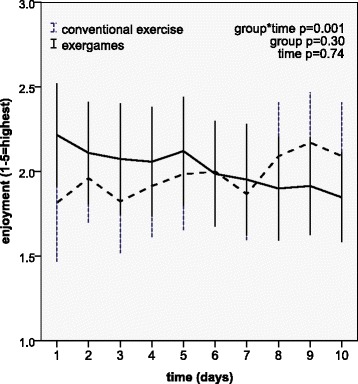
Patient ratings of enjoyment during exercise over 10 days of treatment

Patient ratings of enjoyment during exercise showed a different pattern (Fig. 3), with higher scores in the exergame group on the first day. During the 10 days of treatment enjoyment ratings improved in the conventional group, while they decreased in the exergame group, as shown by the significant interaction between group and time with an effect size of 0.88 (95% CI 0.32–1.44). Effects for time and group were not significant.

**Fig. 4:**
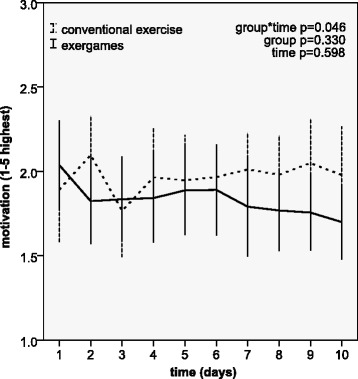
Patient ratings of motivation during exercise over 10 days of treatment

Motivation (Fig. 4) showed a similar pattern, with a slightly higher initial rating in the exergame group. The improvement in the conventional exercise group compared with the exergame group was significant, as shown by the significant interaction between group and time and the moderate effect displayed by an effect size of 0.59 (95% CI 0.05–1.14). There were no effects of group and time.

Balance during walking, measured with an accelerometer, remained unchanged (mean Lyapunov conventional exercise 0.039 (95% CI –0.014 to 0.092), exergame –0.036 (95% CI –0.128 to 0.057), difference 0.075, *p* = 0.16).

### Analysis of available data

Using available data did not essentially change the results of the mixed model analysis of repeated measurements. The benefit of conventional exercise compared with exergames during the treatment phase, expressed by the interaction between group and times, was slightly smaller.

## Discussion

### Main results

This study compared the effect on adherence, motivation, enjoyment and balance abilities of conventional self-regulated exercises with exergames older people in a geriatric rehabilitation setting. Contrary to our expectations, no benefit was found for self-regulated exergames compared with self-regulated conventional exercises regarding adherence, measured as daily training volume. Exercise time and frequency of exercise sessions on the first self-regulated training day were comparable in both groups, but changed significantly over the 10-day period in favor of self-regulated conventional exercises. The significant difference in training volume over the 10-day intervention period between both groups was accompanied by a significant decrease in motivation and enjoyment in the self-regulated exergames group compared with the self-regulated conventional exercises group. There was no group effect on objective balance skills, measured with the accelerometer.

### Strengths of this study

To our knowledge, this is the first study to compare exergames with conventional self-regulated exercises in persons over 65 years of age in a rehabilitation setting. The only available randomized controlled trial [28] compared exercises at home and at a medical center, focusing on usability, user experience and user acceptance. Other available studies focus on older adults, either with brain injury or with cognitive impairment, living in the community. Although recruitment was more than 50% below expectations, the effects were larger than expected and were therefore significant (Cohen’s d > 0.5). In addition, by making repeated measures over a period of ten consecutive days the power of between-group statistical analyses was increased over that of comparing two measurements before and after a treatment phase.

### Limitations

We did not evaluate frailty at baseline. A higher level of frailty in the exergame group might have contributed to the higher drop-out rate in this group. However, groups were comparable for age, diagnosis, multimorbidity, cognition, balance and fear of falling (Table 1). Frailty is associated with several of these baseline measures. We therefore think it is very unlikely that frailty was higher in the exergame group and explained the higher drop-out rate.

A further limitation of the study design is the short duration of the intervention (ten working days). This limitation is due to the length of hospitalization (2 to 3 weeks). Adherence to training for elderly people must be long lasting to be effective. The short study duration, together with comparable co-interventions during rehabilitation in both groups, may explain the absence of an effect on mobility assessed with the ActiGraph^®^ accelerometer. Despite the short duration of the intervention, repeated measures of adherence, motivation and enjoyment gave valuable information, including significant treatment effects.

The use of a self-report logbook to measure daily training volume can be considered a further limitation of the design. A systematic error in self-reporting cannot be excluded, but, as participants in both treatment groups used the same logbook, we assume that such error was comparable in the two groups.

### Recruitment, drop-outs and the proportion of patients disliking exergames

Important limitations of this study are the low recruitment and high drop-out rate, both associated with a considerable proportion of patients disliking exergames. Twenty-one percent of the eligible patients declined to participate because they disliked exergames. Disliking exergames was also associated with a markedly higher dropout rate in the exergame group than in the conventional exercise group. Today’s elderly people seem to prefer paper-based instructions for self-regulated conventional exercise rather than computer-based exergames. These findings suggest a limited clinical use of game-based technology within the geriatric rehabilitation setting.

### Exergames

Within eHealth interventions the exponential decrease in adherence has been described as the “Law of Attrition” [29], and strategies to maximize patient engagement, such as gamification, are being incorporated into eHealth solutions in order to reduce attrition. The exergames used in this study may not have met the maximal standard. During the treatment phase and analysis several points needing attention in future development were noted. Firstly, GameUp was a fully functional prototype, but not the final product. GameUp fulfilled all predefined usability criteria. Initial motivation for self-regulated exercise was equal in the exergame and conventional exercise groups. Patients in the exergames group initially experienced significantly more enjoyment than the patients performing conventional self-regulated exercises, which indicates that the exergames fulfilled initial expectations. Computer handling problems among elderly game players can be ruled out as reason for declining adherence in the exergame group, since a therapy assistant was always present to provide help if needed. However, cognitive demands during exergaming were probably higher in this aged population, limiting the flow experience. The level of social interaction among participants during the scheduled exercise time differed between groups. Patients performed their conventional self-regulated exercises in groups in the same room twice a day, while the exergames group performed their scheduled exercise sessions in a separate room on their own. In the game used in the current study, players were not able to collect points over time. Exercising with others and collecting points are often used to motivate players to play repeatedly. Finally, the setting of this study may have hampered the motivational benefits of exergaming. The use of exergames in the controlled study environment reduced social motivation, which is inherently expected in the game.

### Further research

It is of paramount importance to undertake more implementation research in order to facilitate the translation of eHealth research into clinical practice. Research should identify barriers and facilitators for the implementation of exergames [30, 31]. In cooperation with a group of elderly users participating in the user-centred design and development in Norway, many adaptations were made to make the game easier to understand and more rewarding to play. A social component was added, allowing play in a group with a helper at hand. Players also preferred shorter durations of each mini-game, and played several rounds for 1–1.5 h/session [32]. The same principle is applied in Pokémon Go, in which users can meet at landmarks with many Pokémons. Although this game is clearly not appropriate for our target population, several of its ideas could usefully be adapted [33].

## Conclusions

In a geriatric rehabilitation setting, conventional self-regulated exercise using printed instruction leaflets were superior to self-regulated exercise using exergames with respect to adherence, enjoyment and motivation. Computer-based exergames were initially associated with higher motivation and enjoyment, but this difference reversed over a period of 2 weeks. Balance skills during walking measured with accelerometers remained unchanged in both groups. Improvements in exergames, and randomized controlled trials evaluating their effectiveness compared with other methods of providing self-regulated exercise, are needed before general use in a geriatric rehabilitation setting can be considered.

## Abbreviations

BBS: Berg Balance Scale
CI: Confidence Interval
MMSE: Mini-Mental State Examination
SD: Standard deviation
SPSS: Statistical Package for the Social Sciences
WHO: World Health Organization
λS: Largest Lyapunov exponent
